# GMO discussion on Twitter

**DOI:** 10.1080/21645698.2023.2241160

**Published:** 2023-08-01

**Authors:** Dmitry Erokhin, Nadejda Komendantova

**Affiliations:** Advancing Systems Analysis, International Institute for Applied Systems Analysis, Laxenburg, Austria

**Keywords:** Conspiracy, GMO, misinformation, sentiment analysis, Twitter

## Abstract

This paper focuses on analyzing discussions related to Genetically Modified Organisms (GMOs) on Twitter, with a specific focus on the spread of misinformation and conspiracy theories. The authors collected and analyzed 1,048,274 English tweets related to GMOs between January 2020 and December 2022 using the Twitter API. The tweets were subjected to topical and sentiment analysis to identify the prevalent themes and attitudes toward GMOs. 30.92% of the tweets in the observed period were negative, 21.65% were neutral, and 47.43% were positive. The authors identified four clusters of tweets associated with misinformation or conspiracy theories: GMOs and vaccines, GMOs and COVID-19, GMOs and Monsanto, and GMOs and Bill Gates. The findings of this analysis can inform strategies for combating the spread of false information and conspiracies on social media and improve public understanding and trust in GMO technology.

## 1. Introduction

Genetically Modified Organisms (GMOs) have been the subject of intense public debate and controversy for many years.^1^ While proponents of GMOs argue that they can improve crop yields, enhance nutritional value, and even help combat world hunger, opponents have raised concerns about their safety and long-term effects on the environment and human health. One significant challenge to the public understanding of GMOs is the pervasive spread of misinformation and conspiracy theories surrounding the technology.^2,3^ Under misinformation we understand “false or inaccurate information that is deliberately created and is intentionally or unintentionally propagated,”^4^ whereas a conspiracy theory refers to “explanatory beliefs of how multiple actors meet in secret agreement in order to achieve a hidden goal that is widely considered to be unlawful or malevolent.”^5^

Misinformation regarding GMOs has spread rapidly through social media, anti-GMO advocacy groups, and alternative health websites.^6–8^ Such misinformation often distorts the scientific facts and exaggerates the risks of GMOs, leading to unwarranted public fear and skepticism.^9^ Furthermore, the proliferation of conspiracy theories surrounding GMOs has led to a further erosion of public trust in the scientific community and regulatory agencies.^10^

Conspiracy theories about GMOs include beliefs that GMOs are part of a plot by corporations to control the global food supply^11^ or that they are part of a larger scheme to depopulate the world by causing different diseases (e.g., cancer).^12–14^ Such conspiracies often ignore the rigorous scientific testing and regulatory procedures that GMOs undergo before being approved for human consumption. Alternative health and pro-conspiracy sites receive more social media engagements than mainstream media outlets, highlighting ongoing controversy and the emergence of “non-GMO” marketing approaches.^15^ The spread of misinformation and conspiracies surrounding GMOs can have significant consequences for public policy, scientific research, and public health,^16^ e.g., leading to the facilitation of anti-vaccination behavior.^17^ Misinformation and conspiracy theories can potentially harm public perception and increase hesitancy toward GMO consumption. However, it is important to recognize the numerous benefits associated with GMOs,^18^ which include: increased crop yields, leading to higher productivity and improved agricultural output; enhanced nutritional value by modifying crops to contain higher levels of essential vitamins, minerals, or beneficial compounds; improved crop quality, including enhanced flavor, texture, and shelf life; reduced pesticide use through the development of pest-resistant GMOs, minimizing the environmental impact and potential harm to human health; enhanced tolerance to environmental stressors such as drought, salinity, and extreme temperatures, allowing crops to thrive in challenging conditions; increased food production efficiency by optimizing nutrient uptake and resource utilization; disease resistance in crops, protecting them from devastating pathogens and reducing yield losses; promotion of sustainable agriculture practices by reducing the need for chemical insecticides and supporting more environmentally friendly farming methods.

To gain a more in-depth understanding of public opinion about GMOs, this paper utilizes Twitter data from January 2020 to December 2022. Twitter is an ideal platform for studying public perceptions and attitudes toward GMOs as it is one of the most widely used social media platforms, and users often engage in discussions on various topics.

Using the Twitter API, we collected and analyzed tweets related to GMOs during the specified time period. The collected tweets were then subjected to content analysis to identify the prevalent themes, and sentiment.

By leveraging the power of data analysis, we aim to provide a data-driven perspective on the spread of misinformation and conspiracy theories surrounding GMOs on Twitter. The results of this analysis can inform strategies for combating the spread of false information and conspiracies on social media and improve public understanding and trust in GMO technology. This paper seeks to provide an overview of the current state of the GMO debate and the role of misinformation and conspiracy theories in shaping public opinion.

The paper is structured as follows. Section 2 details the methods and data utilized in the study. Section 3 presents the results. Section 4 provides a comprehensive discussion. Finally, in Section 5, the paper concludes with a summary of the findings.

## 2. Methods and Data

To collect relevant data, we used the Twitter Academic API, which provides access to a large sample of tweets in real-time. We used the keyword “GMO” to filter tweets that specifically discussed genetically modified organisms. We selected the search term “GMO” specifically to focus on the most commonly used and recognized term related to genetically modified organisms. This decision was driven by the need for consistency and clarity in the data collection process. While alternative terms such as “genetically modified food” or “GM food” could have been included, we expected that the term “GMO” would capture a significant portion of the relevant tweets and discussions on social media. We limited our search to English language tweets. Each tweet included in the data set contained at least one occurrence of the keyword “GMO” in the tweet text. We collected all the GMO-related English tweets between January 2020 and December 2022.

To determine the sentiment of the collected tweets, we used Microsoft Azure Sentiment Analysis. It provides a sentiment score for each tweet between 0 and 1, where 0 represents negative sentiment and 1 represents positive sentiment. We received sentiment scores for each tweet and calculated mean sentiment for each month of the observed period. Sentiment analysis allowed us to understand the general attitude toward GMOs on Twitter during the observed period. By analyzing the mean sentiment for each month, we were able to identify any changes in sentiment over time. However, it is crucial to acknowledge the limitations associated with sentiment analysis in this context. Specifically, the model employed in this study was trained to predict sentiment for products and services rather than to assess discussions on complex subjects like GMOs. This mismatch in training data may impact the accuracy and applicability of sentiment analysis results when applied to GMO-related discussions. Hence, while sentiment analysis offers insights into the overall sentiment trend, caution should be exercised in interpreting and generalizing the findings, considering this inherent limitation. Nevertheless, there are studies, which have successfully applied this methodology to various contexts e.g.^19–21^

To conduct the topical analysis of the tweets, we determined the frequency of different words in the tweet text, excluding stop words such as “the,” “and,” etc. We then analyzed the top 10 most frequent words that had a meaning and could provide insight into the topics of discussion related to GMOs on Twitter. We used this method to identify the most common topics discussed by Twitter users in relation to GMOs. We also conducted further analysis of the most frequent words to identify any trends or patterns in the topics discussed.

Moreover, we examined four clusters of tweets that we presume are closely associated with misinformation or conspiracy theories. These clusters pertain to the link between GMO and vaccines,^3^ the connection between GMO and COVID-19,^13^ the association between GMO and Monsanto,^22^ and the relationship between GMO and Bill Gates.^22^ All four topics have a significant GMO-related aspect. These topics have been the subject of significant discussions on social media platforms due to the perceived benefits and risks of GMOs. In addition to this, all four topics have also been the subject of misinformation and conspiracies related to GMOs. For example, there have been claims that the COVID-19 vaccines contain microchips or other tracking devices, which has been widely debunked. Similarly, Monsanto has been the subject of numerous conspiracy theories, including claims that their products are responsible for cancer and other health issues. Bill Gates has also been the target of numerous conspiracy theories, many of which are related to his work on GMOs and global health initiatives. These theories range from claims that he is trying to depopulate the world through vaccines to accusations that he is using GMOs to control the world’s food supply.

We have to note that active measures taken by Twitter to combat misinformation during the pandemic may have influenced the amount of misinformation available, potentially reducing its prevalence and impact on the study’s findings.

## 3. Results

**Figure 1.**
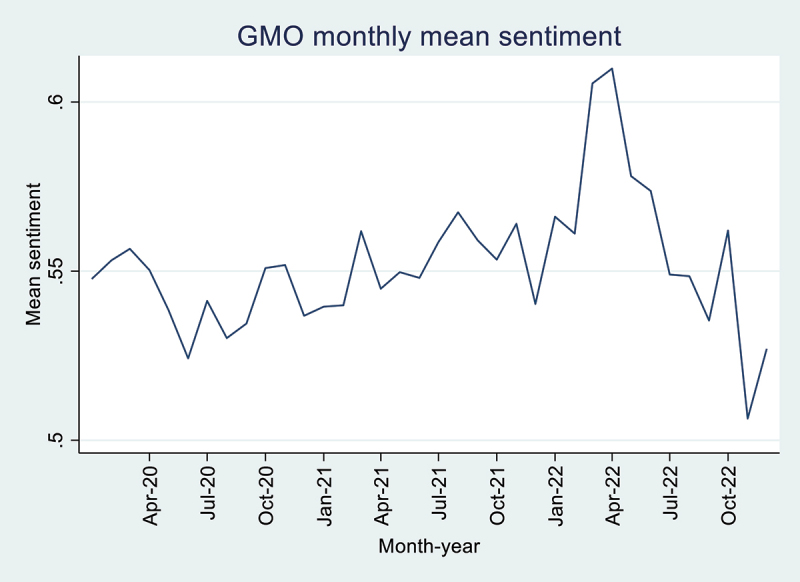
GMO monthly mean sentiment.

**Figure 2.**
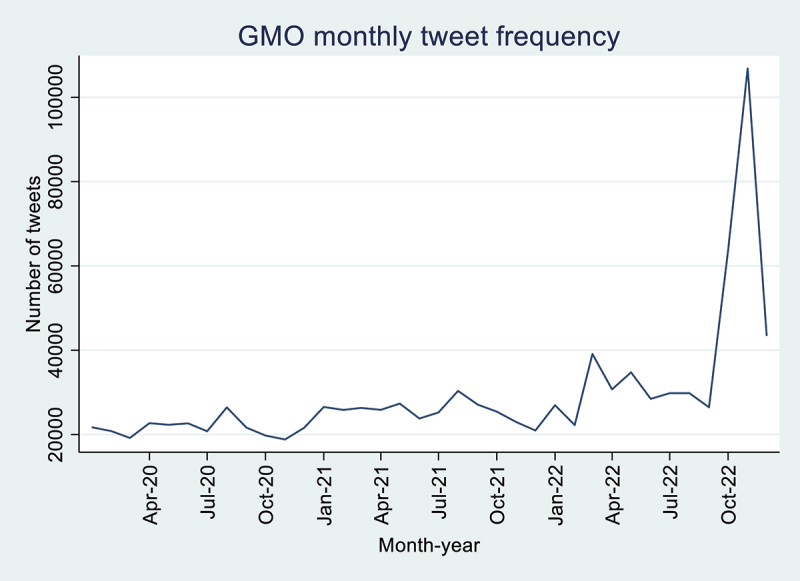
GMO monthly tweet frequency.

In total, we have 1,048,274 English tweets on GMO from January 2020 to December 2022. Figure 1 shows mean sentiment for each month from January 2020 to December 2022. The mean sentiment ranges from 0.5064 to 0.6099, with the highest mean sentiment occurring in March 2022 and April 2022 and the lowest occurring in November 2022. The tweet frequency (see Fig. 2) ranges from 18,825 to 106,856, with the highest tweet frequency occurring in November 2022 and the lowest occurring in January 2020. We find that 30.92% of the tweets in the observed period were negative, 21.65% were neutral, and 47.43% were positive.

The topic analysis (see Table A1) reveals that GMOs were frequently discussed on Twitter between January 2020 and December 2022. The most commonly used words across all the months include “Non,” “Free,” “Food,” “Organic,” and “Crops.” Some tweets suggest that people are concerned about the impact of GMOs on health, the environment, and food security. Other tweets show support for GMOs, arguing that they are necessary to meet the growing demand for food. There are also mentions of Monsanto, a multinational agricultural biotechnology company, with some tweets suggesting that they are behind the push for GMOs. The ongoing and lively discussion surrounding Monsanto as a prominent topic in GMO discourse for a significant portion of the observed months becomes even more intriguing when considering the fact that Monsanto ceased to exist as a company in 2018. However, it seems that Monsanto continues to play a significant role in the GMO discourse between 2020 and 2022.

Additionally, there are some conspiracy theories and misinformation about GMOs on Twitter. Some tweets suggest that GMOs cause health problems such as cancer, while others argue that they are part of a plan to control the world’s food supply. There are also mentions of Bill Gates and his supposed involvement in promoting GMOs. Finally, some tweets discuss the use of genetically modified mosquitoes to control disease, with some expressing concerns about the safety and effectiveness of this approach. Overall, the topic of GMOs is complex and controversial, with different opinions and viewpoints being expressed on Twitter.

We looked separately at the four clusters of tweets, which we assume are related to the discussion on misinformation and conspiracy theories, i.e., GMO and Bill Gates, GMO and vaccines, GMO and COVID-19 as well as GMO and Monsanto (see Table A2). Overall, the presence of misinformation and conspiracies related to GMOs and these four topics has further fueled discussions on social media platforms. Between January 2020 and December 2022, we had 29,072 tweets related to GMO and COVID-19, 44528 tweets related to GMO and Bill Gates, 43816 tweets related to GMO and Monsanto, and 59,931 tweets related to GMO and vaccines. The COVID-19 topic had a mean sentiment of 0.5437, the vaccines topic had a mean sentiment of 0.5637, the Monsanto topic had a mean sentiment of 0.5534, and the Bill Gates topic had a mean sentiment of 0.4943. The Bill Gates related tweets had the highest share of negative tweets among all tweets analyzed − 43.50%. In the COVID-19 topic, people discussed the vaccine and its development, as well as its potential impact on health. The top 10 frequent words suggest that people were concerned about how mRNA technology used in the vaccine could be applied and whether it would be safe. The vaccines topic was dominated by discussions around mRNA and biochemistry. People were also talking about the potential benefits and risks of vaccines, with specific references to mosquitoes and experimental vaccines. Monsanto was the top frequent word in discussions about the company. People talked about their products such as Roundup, and the safety of GMOs, particularly related to crops like corn and seeds. The role of farmers in producing GMOs was also mentioned. Finally, the Bill Gates topic had a more varied discussion with references to African food, videos, and lying. The top 10 frequent words suggest that people were concerned about the impact of GMOs on African food and agriculture, as well as the potential for people to be misled or uninformed about the safety of GMOs.

## 4. Discussion

The findings of this study shed light on the complex relationship between societal perception and established science regarding GMOs, particularly on social media platforms like Twitter. The analysis of collected tweets reveals valuable insights into public opinion and the contrast between scientific fact and societal perception.

Over decades of research and assessments, the safety of genetically modified crops has garnered strong support from the global scientific community.^23–25^ The prevailing scientific consensus affirms that foods and feeds derived from GMOs are as safe and nutritious as those derived through conventional breeding techniques. This consensus is based on rigorous scientific testing and evaluations.

However, the study uncovers a significant contrast between this scientific consensus and public opinion on social media platforms like Twitter. While scientific evidence supports the safety of GMOs, public sentiment diverges greatly from this consensus.^26–28^ The sentiment analysis of collected tweets reflects mixed attitudes toward GMOs, influenced by various factors including misinformation and conspiracy theories that circulate on social media. The share of negative sentiment we identify is close to the results by Sohi et al.^29^ approximately one third of the tweets. However, we have varying shares of neutral and positive tweets, which can be due to differences in the underlying sentiment analysis models.

The prevalence of misinformation and conspiracy theories surrounding GMOs on Twitter is a major concern. These narratives distort scientific facts, amplify risks, and contribute to unwarranted fear and skepticism among the public. False narratives range from claims of GMOs being part of a corporate conspiracy to control the global food supply to accusations of depopulation schemes. These conspiracies overlook the rigorous testing and regulatory procedures that GMOs undergo before approval.

To delve deeper into the GMO controversy, it is crucial to explore how societal perception is at odds with established science. This involves understanding the reasons behind public skepticism, concerns, and misinformation. By examining the prevalent themes and attitudes on social media, we can identify knowledge gaps and misconceptions that need to be addressed through targeted interventions.

The study highlights the importance of combatting the spread of misinformation and conspiracy theories on social media platforms. Scientific institutions, regulatory agencies, and GMO advocates have a responsibility to actively engage with the public, debunk myths, and provide accurate scientific information. Effective communication strategies that bridge the gap between scientific consensus and public perception are vital in fostering informed decision-making and responsible governance.

Furthermore, the results of this study can inform public policy and scientific research related to GMOs. By understanding the prevailing sentiments and themes on social media, policymakers can develop initiatives that address misconceptions and concerns, and promote accurate information about GMOs. Public education campaigns and transparent policy initiatives can contribute to a better alignment between public perception and established science.

The study also reveals additional insights into GMO public opinion on Twitter. The analysis of the collected tweets highlights the prevalence of misinformation and conspiracy theories surrounding GMOs, particularly in clusters associated with vaccines, COVID-19, Monsanto, and Bill Gates. These topics fuel the dissemination of false information and contribute to the divergence between societal perception and scientific consensus. The sentiment analysis demonstrates mixed attitudes toward GMOs, indicating the influence of various factors, including misinformation and conspiracy theories, on public sentiment. Additionally, the topic analysis provides further insights into the nature of discussions, reflecting concerns about health, the environment, and food security, as well as support for GMOs to address increasing food demand.

Overall, the findings underscore the need for strategies to combat misinformation and conspiracy theories, improve public understanding, and bridge the gap between scientific fact and societal perception on GMOs. By addressing false narratives, engaging with the public, and promoting accurate scientific information, we can foster a more informed and constructive dialogue on GMOs and contribute to responsible decision-making and governance.

## 5. Conclusion

This paper aimed to provide insights into the role of misinformation and conspiracy theories in shaping public opinion about GMOs on Twitter. The prevalence of misinformation and conspiracy theories surrounding GMOs is a significant challenge to public understanding and trust in GMO technology. The results of the study based on Twitter data analysis reveal that social media platforms such as Twitter are a source of discussion and dissemination of such information, and this misinformation often distorts the scientific facts and exaggerates the risks of GMOs, leading to unwarranted public fear and skepticism. Through the analysis of sentiment and content, the study identified the most frequent topics discussed in relation to GMOs, including the link between GMO and vaccines, the connection between GMO and COVID-19, the association between GMO and Monsanto, and the relationship between GMO and Bill Gates. These topics have been the subject of significant discussions on social media platforms and are also associated with misinformation and conspiracies related to GMOs. The study highlights the need for strategies to combat the spread of false information and conspiracies on social media, improve public understanding and trust in GMO technology, and inform public policy and scientific research related to GMOs.

## Appendix

**Table A1.** Tweet frequency, mean sentiment, and top 10 frequent words on GMO between January 2020 and December 2022.

Month year	Tweet frequency	Negative/neutral/positive sentiment	Mean sentiment (standard deviation)	Top 10 frequent words
January 2020	21,729	31.17% 22.96% 45.86%	0.5477 (0.2403)	Non (3229) Free (2346) Food (2043) Organic (2012) Meat (1466) New (1039) Crops (1034) Monsanto (1019) People (972) Foods (920)
February 2020	20,804	25.92% 34.10% 39.98%	0.5532 (0.2166)	Non (5038) Homegrown (2867) Free (1737) Food (1731) Organic (1428) Crops (1005) Monsanto (954) Foods (753) People (719) Seeds (707)
March 2020	19,188	28.11% 24.95% 46.94%	0.5566 (0.2204)	Non (2809) Free (1993) Organic (1936) Food (1794) Crops (1618) Health (1063) New (1043) Foods (853) Trump (838) Coronavirus (815)
April 2020	22,704	30.99% 22.75% 46.26%	0.5503 (0.2309)	Non (2811) Food (2564) Free (2083) Organic (1687) Monsanto (1566) Seeds (1200) Crops (1113) Foods (941) People (932) Health (858)
May 2020	22,305	31.84% 25.03% 43.12%	0.5385 (0.2288)	Non (2400) Food (2222) Organic (1591) Free (1543) Monsanto (1206) New (1050) Crops (993) Gates (931) Natural (901) Foods (845)
June 2020	22,648	36.45% 23.93% 40.52%	0.5242 (0.2323)	Food (2182) Non (2167) Meat (1985) Chicken (1750) Organic (1665) Beef (1647) Never (1561) Animals (1526) Free (1525) Hormone (1510)
July 2020	20,777	32.52% 21.88% 45.61%	0.5412 (0.2398)	Non (2603) Food (2415) Free (1829) Organic (1412) New (1330) Foods (1304) Crops (1151) Monsanto (922) People (853) Health (834)
August 2020	26,436	30.30% 29.81% 39.89%	0.5302 (0.2237)	Mosquitoes (4122) Genetically (3753) Release (3265) Modified (2799) Non (2574) Florida (2490) Food (2267) Plan (2060) 2021 (2057) Free (2032)
September 2020	21,644	34.62% 19.85% 45.52%	0.5345 (0.2346)	Non (2294) Food (2012) Free (1707) Crops (1625) Organic (1380) New (1059) World (956) Beef (940) People (929) Corn (885)
October 2020	19,760	30.96% 22.77% 46.27%	0.5509 (0.2291)	Food (2363) Non (2019) Free (1487) Organic (1459) Foods (1101) Wheat (1040) Monsanto (939) Crops (917) Corn (813) Seeds (791)
November 2020	18,825	30.39% 21.48% 48.13%	0.5518 (0.2373)	Food (2500) Non (2332) Free (1414) Organic (1269) Corn (1134) Foods (1010) New (895) Monsanto (836) Health (809) People (791)
December 2020	21,592	30.84% 24.84% 44.33%	0.5368 (0.2379)	Non (2674) Food (2670) Corn (2144) Free (1350) Organic (1297) Real (1248) New (1213) Monsanto (1174) Public (1085) Vaccine (1065)
January 2021	26,535	34.03% 20.83% 45.14%	0.5395 (0.2358)	Food (4034) Non (2886) Organic (2331) Corn (2328) People (1476) Free (1456) Mexico (1373) Crops (1298) Genetically (1239) New (1191)
February 2021	25,839	34.62% 18.66% 46.72%	0.5399 (0.2443)	Food (3122) Non (2446) Free (2110) Genetically (1825) Eat (1511) Corn (1469) Mrna (1433) Organic (1428) Modified (1416) Crops (1381)
March 2021	26,313	30.73% 20.49% 48.78%	0.5618 (0.2414)	Food (3272) Non (2501) Vaccines (2307) Free (1844) Organic (1718) Corn (1527) Billions (1356) Mass (1354) Seeds (1343) Pandemics (1315)
April 2021	25,857	30.00% 25.08% 44.91%	0.5448 (0.2369)	Food (3156) Non (2375) People (1889) Mosquitoes (1796) Free (1568) New (1561) Organic (1549) Florida (1532) Vaccine (1515) Crops (1320)
May 2021	27,339	30.39% 21.20% 48.51%	0.5497 (0.2308)	Food (3249) Non (3193) Mosquitoes (2769) Organic (1909) Release (1640) Corn (1637) Free (1604) People (1571) Billion (1382) Gates (1356)
June 2021	23,799	29.53% 20.78% 49.68%	0.5480 (0.2501)	Food (3254) Real (3007) Non (2388) Experimental (1855) Trials (1788) People (1748) Event (1720) Vaccination (1706) Requiring (1684) Concoction (1683)
July 2021	25,229	29.98% 20.19% 49.83%	0.5586 (0.2367)	Non (2719) Food (2578) Organic (1976) Corn (1786) Foods (1678) Crops (1588) Free (1551) People (1307) Genetically (1173) Monsanto (1144)
August 2021	30,350	31.21% 14.75% 54.04%	0.5674 (0.2357)	Meat (2649) Food (2626) Non (2531) Organic (2044) Day (1945) Fake (1886) Impossible (1854) Burger (1833) Concerns (1820) Safety (1817)
September 2021	27,105	26.16% 24.14% 49.70%	0.5591 (0.2249)	Non (2634) Food (2589) Organic (1939) Free (1591) Foods (1386) People (1335) Crops (1198) Eat (1065) Corn (1062) New (922)
October 2021	25,433	29.50% 19.67% 50.82%	0.5534 (0.2290)	Non (2961) Food (2478) Corn (2051) Organic (1963) Free (1804) Foods (1390) New (1183) People (1057) Crops (1018) Mexico (920)
November 2021	22,946	29.17% 21.43% 49.40%	0.5640 (0.2303)	Food (2534) Organic (2453) Non (2204) Free (1709) People (1123) Crops (1122) Corn (1109) Foods (1086) Green (1027) Human (939)
December 2021	20,952	33.32% 19.27% 47.41%	0.5403 (0.2417)	Non (3124) Food (2663) Organic (2080) Free (1490) Foods (1139) People (1020) Corn (890) Seeds (813) Monsanto (801) Comply (798)
January 2022	26,955	28.57% 20.39% 51.04%	0.5661 (0.2467)	Food (4465) Non (2826) Foods (2288) New (2260) Organic (2017) Free (1920) Labeling (1425) Modified (1382) Corn (1354) People (1315)
February 2022	22,251	30.49% 19.17% 50.34%	0.5611 (0.2329)	Food (2597) Non (2377) Organic (1735) Free (1420) Foods (1214) Seed (1191) Corn (1191) People (1137) Crops (969) Sugar (845)
March 2022	39,118	26.01% 17.65% 56.33%	0.5903 (0.2320)	Food (5980) Know (3755) Corn (3648) Time (3456) Labeling (3434) Mosquitoes (3258) Countries (3207) Demand (2965) Local (2941) Require (2940)
April 2022	30,719	23.05% 17.18% 59.78%	0.6099 (0.2333)	Know (4895) Time (4657) Labeling (4449) Countries (4377) Demand (4223) Require (4214) Food (3711) Non (2697) Free (1821) Big (1700)
May 2022	34,759	26.35% 21.61% 52.04%	0.5781 (0.2299)	Food (4969) Foods (4902) Non (4588) Know (3725) Time (3254) Countries (3244) Labeling (2928) Demand (2847) Require (2799) Organic (1866)
June 2022	28,463	27.24% 20.31% 52.45%	0.5737 (0.2295)	Non (3752) Food (3227) Gates (2529) Crops (2244) Time (1795) Labeling (1600) Foods (1591) Countries (1583) Free (1525) Require (1451)
July 2022	29,810	30.15% 21.47% 48.38%	0.5490 (0.2328)	Non (4817) Food (3777) Organic (2745) Free (2275) Corn (2124) Seeds (1607) Crops (1534) Farmers (1507) Wheat (1353) Foods (1274)
August 2022	29,815	30.23% 21.45% 48.32%	0.5485 (0.2329)	Non (4807) Food (3777) Organic (2762) Free (2276) Corn (2134) Seeds (1595) Crops (1539) Farmers (1521) Wheat (1354) Foods (1277)
September 2022	26,461	33.62% 19.66% 46.72%	0.5354 (0.2389)	Non (4263) Food (3577) Corn (2267) Organic (2006) Free (1699) Human (1607) Soy (1568) People (1221) Crops (1197) Foods (1123)
October 2022	63,531	26.67% 22.96% 50.37%	0.5620 (0.2240)	Food (9661) Project (7801) Proof (5763) Kenya (5214) Ban (4717) Seeds (4366) Foods (4085) Reply (3973) Non (3392) Hours (3241)
November 2022	106,856	38.41% 21.06% 40.53%	0.5064 (0.2420)	Maize (17622) Food (12361) Kenya (12115) Gates (9748) Bill (9073) Foods (7310) Non (7253) Kenyans (6668) Kuria (6037) Country (5992)
December 2022	43,427	35.55% 22.96% 41.49%	0.5270 (0.2271)	Non (9127) Free (5674) Food (5097) Foods (4086) Organic (3456) Corn (3082) Gluten (2915) Vegan (2614) Seeds (2245) Amazon (1889)

**Table A2.** Tweet frequency, mean sentiment, and top 10 frequent words on four GMO-related topic clusters between January 2020 and December 2022.

Topic	Tweet frequency	Negative/neutral/positive sentiment	Mean sentiment (standard deviation)	Top 10 frequent words
COVID-19	29,072	30.73% 23.69% 45.58%	0.5437 (0.2259)	Vaccine (4579) Gates (3009) Food (2376) Vaccines (2325) Crops (2005) People (1871) Mrna (1586) Bill (1522) Tanzania (1414) Health (1367)
Vaccines	59,931	28.10% 18.54% 53.36%	0.5637 (0.2366)	Mrna (14225) Biochem (9928) Food (7198) Gates (5225) COVID (4531) People (4183) Bill (3575) Humans (3377) Mosquitoes (3177) Experimental (3016)
Monsanto	43,816	31.33% 22.93% 45.75%	0.5534 (0.2164)	Bayer (6452) Food (6245) Corn (5193) Seeds (5097) Gates (3178) Farmers (2825) Roundup (2775) Crops (2649) Safety (2618) Glyphosate (2391)
Bill Gates	44,528	43.50% 16.83% 39.67%	0.4943 (0.2455)	Food (7014) African (4139) Video (3822) Bread (3662) Eaten (3585) Lying (3498) Caught (3235) Mosquitoes (3172) Audience (3163) Eat (3072)
